# Antiatherosclerotic Effect of Korean Red Ginseng Extract Involves Regulator of G-Protein Signaling 5

**DOI:** 10.1155/2014/985174

**Published:** 2014-12-24

**Authors:** Eun Ju Im, Taddesse Yayeh, Sang-Joon Park, Seung-Hyung Kim, Youn-Kyoung Goo, Seung-Bok Hong, Young Min Son, Sung Dae Kim, Man Hee Rhee

**Affiliations:** ^1^Laboratory of Veterinary Physiology and Cell Signaling, College of Veterinary Medicine, Kyungpook National University, Daegu 702-701, Republic of Korea; ^2^Department of Animal Science, College of Agriculture and Natural Resource, Debre Markos University, P.O. Box 269, Debre Markos, Ethiopia; ^3^Laboratory of Veterinary Histology, College of Veterinary Medicine, Kyungpook National University, Daegu 702-701, Republic of Korea; ^4^Institute of Traditional Medicine & Bioscience, Daejeon University, Daejeon 300-716, Republic of Korea; ^5^Department of Parasitology and Tropical Medicine, Kyungpook National University School of Medicine, Daegu 700-422, Republic of Korea; ^6^Department of Clinical Laboratory Science, Chungbuk Health & Science University, Chungbuk 363-794, Republic of Korea; ^7^Research Center, Dongnam Institute of Radiological and Medical Sciences, Busan 619-953, Republic of Korea

## Abstract

Regulator of G-protein signaling 5 (RGS5), an inhibitor of G*α*(q) and G*α*(i) activation, has been reported to have antiatherosclerosis. Previous studies showed antiatherosclerotic effect of Korean red ginseng water extract (KRGE) via multiple signaling pathways. However, potential protective effect of KRGE through RGS5 expression has not been elucidated. Here, we investigated the antiatherosclerotic effect of KRGE *in vivo* and *in vitro* and its role on RGS5 mRNA expression. Elevated levels of total cholesterol, lactate dehydrogenase (LDH), and triglyceride (TG) in western diet groups of low-density lipoprotein receptor deficient LDLr^−/−^ mice were reversed by oral administration of KRGE. KRGE suppressed transcriptional activity of tumor necrotic factor alpha (TNF-*α*), interleukin-6 (IL-6), and leptin in adipose tissue. It also potently repressed western diet-induced atheroma formation in aortic sinus. While KRGE showed reduced mRNA expression of inducible nitric oxide synthase (iNOS), cyclooxygenase-2 (COX-2), IL-1*β*, IL-6, and TNF-*α* in LPS-stimulated RAW264.7 cells, it enhanced mRNA expression of RGS5. Moreover, RGS5 siRNA transfection of microglia cells pretreated with KRGE reversed its inhibitory effect on the expression of iNOS, COX-2, and IL-1*β* mRNA. In conclusion, KRGE showed antiatherosclerotic and anti-inflammatory effects in western diet fed LDLr^−/−^ mice and this effect could partly be mediated by RGS5 expression.

## 1. Introduction

Cardiovascular diseases (CVD) have reached epidemic proportion in Western and Asian societies due to urbanization, economic growth, and irregular timing of meals. Few studies also suggested that metabolic disorders may play a significant role in atherosclerosis [1], which is the most common CVD and the primary cause of heart failure and stroke. Atherosclerosis initiated when cholesterol-containing low-density lipoproteins activate the endothelium to express leukocyte adhesion molecules (intracellular adhesion molecule-1 (ICAM-1) [2] and vascular adhesion molecule-1 (VAM-1) [3] and chemokines like macrophage chemoattractant protein-1 (MCP-1) [4]) that promote the recruitment of monocytes and T cells into atherosclerotic prone sites in aortic and carotid arteries [5]. Atherosclerosis begins with subendothelial accumulations of cholesterol-engorged macrophages [6], lipid-rich necrotic debris, and smooth muscle cells [7] altogether forming “fatty streaks” [8]. While low-density lipoprotein (LDL) is associated with the formation of fatty streaks and subsequent plaque formation, high-density lipoprotein (HDL) removes excess cholesterol from peripheral tissues and prevents oxidation of LDL. It is therefore the imbalance of these two lipoproteins in the serum that leads to the development of atherosclerotic lesions at the predilection sites in large arteries [9]. Moreover, adipokines and cytokines are critically involved in the initiation and perpetuation of atherosclerosis [10].

G-protein-coupled receptors (GPCRs) have a critical role in cardiovascular signal transduction, and they could be potential targets for the treatment of CVDs. Regulator of G-protein signaling (RGS) 5, a member of RGS superfamily, exhibits GTPase-activating proteins (GAPs) for *α*-subunit of heterotrimeric G-proteins. It has been known to negatively regulate G-protein coupled receptor (GPCR) mediated signaling pathway (G*α*_i_ and G*α*_q_) through the expression of angiotensin II and endothelin [11].

Korean red ginseng has been used as herbal medicine in Korea, China and Japan. Commercially available ginseng has been used to suppress hepatic steatosis, inhibit platelet activation, relieve pain, attenuate overactivation of macrophages, and protect against ischemia and cancer [12–15]. Previous studies also indicated the activity of RGS5 to regulate cardiac hypertrophy, atherosclerosis, and vascular remodeling. However, potential protective effect of Korean red ginseng water extract (KRGE) through RGS5 expression has been unknown. In this study, therefore, we investigated that KRGE impaired the development of atherosclerosis in LDL receptor deficient (LDLr^−/−^) mice fed with western diet (WD), and this event was involved by RGS5 mRNA expression.

## 2. Materials and Methods

### 2.1. The Preparation of Korean Red Ginseng Water Extract

Korean red ginseng water extract was prepared from roots of a 6-year-old ginseng,* Panax ginseng* C. A. Meyer, harvested in Korea. KRGE was made by steaming fresh ginseng at 90–100°C for 3 h and then drying at 50–80°C. KRGE was prepared from red ginseng water extract, which was extracted at 85~90°C for 8 h of circulating hot water three times. The water content of the pooled extract was 35.7% of total weight. KRGE was analyzed by high-performance liquid chromatography. KRG extract contained major ginsenoside Rb1 (G-Rb1): 7.44 mg/g, G-Rb2: 2.59 mg/g, G-Rc: 3.04 mg/g, G-Rd: 0.91 mg/g, G-Re: 1.86 mg/g, G-Rf: 1.24 mg/g, G-Rg1: 1.79 mg/g, G-Rg2: 1.24 mg/g, G-Rg3: 1.39 mg/g, and other minor ginsenosides.

### 2.2. Materials

Dulbecco's modified Eagle's medium (DMEM) and foetal bovine serum (FBS) were obtained from WelGene (Daegu, Korea). Streptomycin and penicillin were obtained from Lonza (MD, USA). TRI reagent solution (AM9738) and SYBER green master mix were obtained from Applied Biosystems/Ambion (Warrington, UK). Oligo(dT) primers, RT premix, and PCR premix were obtained from Bioneer Co. (Daejeon, Korea). iNOS, COX-2, TNF-*α*, IL-1*β*, IL-6, Leptin, adiponectin, RGS5, and Glyceraldehyde 3-phosphate dehydrogenase (GAPDH) primers were obtained from Bioneer Co. (Table 1) (Daejeon, Korea). Total protein lysis buffer (PRO-PREP) and the PRO-Measure protein assay kit were obtained from iNtRON Biotechnology (Seoul, Korea). LPS (*Escherichia coli* 055:B5) and 3-(4,5-dimethylthiazol-2-yl)-2,5-diphenyltetrazoliumbromide (MTT) were purchased from Sigma (St. Louis, MO, USA). All other reagents and chemicals were obtained from Sigma Aldrich (St. Louis, MO, USA).

### 2.3. Animals

Male LDLr^−/−^ mice, 6 weeks old, were obtained from The Jackson Laboratory (Sacramento, CA, USA) and housed in standard conditions with free access to chow and water and acclimated for 1 week before use. All* in vivo* experiments were conducted in accordance with internationally accepted guidelines in a specific pathogen-free facility, and the protocols were approved by the Institutional Animal Care and Use Committee of Daejeon University. Mice were fed a WD or normal chow. LDLr^−/−^ mice were randomly divided into 5 groups: two normal chow (NC) groups, one WD group, and WD with KRGE 50 mg/kg treatment group or a WD with KRGE 200 mg/kg treatment group (*n* = 6 for each group). LDLr^−/−^ mice were given oral doses of KRGE (50 or 200 mg/kg body weight per day) and then monitored daily for any clinical illnesses. At the end of the 13 weeks, mice were sacrificed. Blood and tissues were collected and stored in aliquots at −80°C until analysis.

### 2.4. Blood Biochemical Analysis

Serum samples were obtained after overnight fasting. LDH, glucose, total cholesterol, HDL, LDL, triglyceride, AST, and ALT were analyzed using enzymatic method (FUJI DRI-CHEM 4000i, FUJI, Japan).

### 2.5. Histological Analysis

The aorta was fixed overnight in 10% formalin solution, dehydrated, embedded in paraffin, and cut into 4 *μ*m section. Cross sections of carotid artery were stained with hematoxylin and eosin (H&E).

### 2.6. RNA Extraction, RT-PCR, and Quantitative PCR

RAW264.7 cells or BV2 microglia were pretreated with or without KRGE at various concentrations for 30 min and then stimulated with LPS (0.1 *μ*g/mL) for 18 h. Total RNA from cell and adipose tissue was extracted using TRI reagent solution (AM9738, Applied Biosystems, Ambion) according to manufacturer instruments. Total RNA (2 *μ*g) was annealed with oligodT (Bioneer Co, Daejeon, Korea) for 10 min at 70°C and cooled for 5 min on ice, reverse-transcribed using reverse transcriptase premix (Bioneer Co, Daejeon, Korea) in 20 *μ*L of reaction mixture, and run for 90 min at 42.5°C using thermal cycler. The reactions were terminated at 95°C for 5 min to inactivate the reverse transcriptase. The reverse transcription polymerase chain reaction (RT-PCR) was performed using aliquots of cDNA obtained from RT reaction in a PCR premix (Bioneer Co, Daejeon, Korea). Quantitative PCR was performing with CFX96 Real-Time System (Bio-Rad, USA) using power SYBER green master mix (Warrington, UK). The relative quantity of target mRNA was calculated using the comparative threshold (Ct) method by GAPDH Ct values. The PCR program is as follows: predenaturation (95°C, 5 min), denaturation (95°C, 20 sec), annealing (55°C, 20 sec), and extension (72°C, 45 sec) using primer specific for GAPDH, iNOS, COX-2, leptin, adiponectin, IL-1*β*, IL-6, and TNF-*α*. The PCR products were electrophoresed in 1.3% agarose gel stained with ethidium bromide and visualized using Eagle Eyes image analysis software (Stratagene, La Jolla, CA). The intensity of band densities was normalized for the corresponding GAPDH, which is housekeeping gene used as an RNA internal standard, and ratios were compared.

### 2.7. Cell Culture

RAW264.7 cells and BV2 microglia cells were maintained in Dulbecco's modified Eagle's medium (DMEM) enriched with 10% heat-inactivated fetal bovine serum (WelGene Co., Daegu, Korea), 100 *µ*g/mL streptomycin, and 100 U/mL penicillin (Lonza, MD, USA) and were incubated in the culture medium in a humidified atmosphere of 5% CO_2_ at 37°C.

### 2.8. Determination of Nitric Oxide Production

Nitric oxide (NO) in cell supernatants was measured by Griess reaction. Briefly, cultured RAW264.7 cells or BV2 microglia in 24-well plates were incubated with or without LPS (0.1 *μ*g/mL) in absence or presence of KRGE at the indicated concentration for 18 h. The cell culture supernatants (100 *μ*L) were mixed with Griess reagent (1% sulphanilamide in 5% phosphoric acid (H_3_PO_4_) and 0.1% N-1-naphthylenediamine dihydrochloride (NEDHC)) in deionized distilled water at equal volume and incubated for 5 min at room temperature. The absorbance of each well was analyzed at 540 nm in ELISA reader.

### 2.9. Cell Viability (MTT) Assay

To check the cytotoxic effect of KRGE, cell viability assay was done using 3-(4,5-dimethyl-thiazol-2-yl)-2,5-diphenyl tetrazolium bromide reagent, which was added to the culture medium at a final concentration of 0.5 mg/mL. After 4 h of incubation at 37°C in 5% CO_2_, the resulting dark blue crystals were dissolved with dimethyl sulfoxide (DMSO, 200 *μ*L) and absorbance values were measured at 560 nm.

### 2.10. siRNA Transfection in BV2 Microglia

BV2 microglia cells were cultured in DMEM with 10% fetal bovine serum and 1% penicillin/streptomycin at 37°C humidified 5% CO_2_. Two different RGS5 siRNA were custom synthesized from Genolution Pharmaceutical, Inc. Lipofectamine 2000 (Invitrogen, Hong Kong) was used as transfection reagent. Transfection was performed according to the manufacturer's instruction. Briefly, BV2 microglia cells were cultured in 24-well plate in DMEM without penicillin/streptomycin. siRNA were used at 100 nM to transfect BV2 microglia using Lipofectamine in antibiotic-free media for 5 h.

### 2.11. Statistical Analysis

The results were presented as mean ± standard error of the mean. One-way analysis of variance followed by Dunnett's *t*-test was used for statistical analysis. *P* values less than 0.05 were considered as statistically significant.

## 3. Results

### 3.1. KRGE Reduced Serum Lipid Profiles and Enzymes Used for Liver Function Tests in LDLr^−/−^ Mice Fed with Western Diet

**Figure 1:**
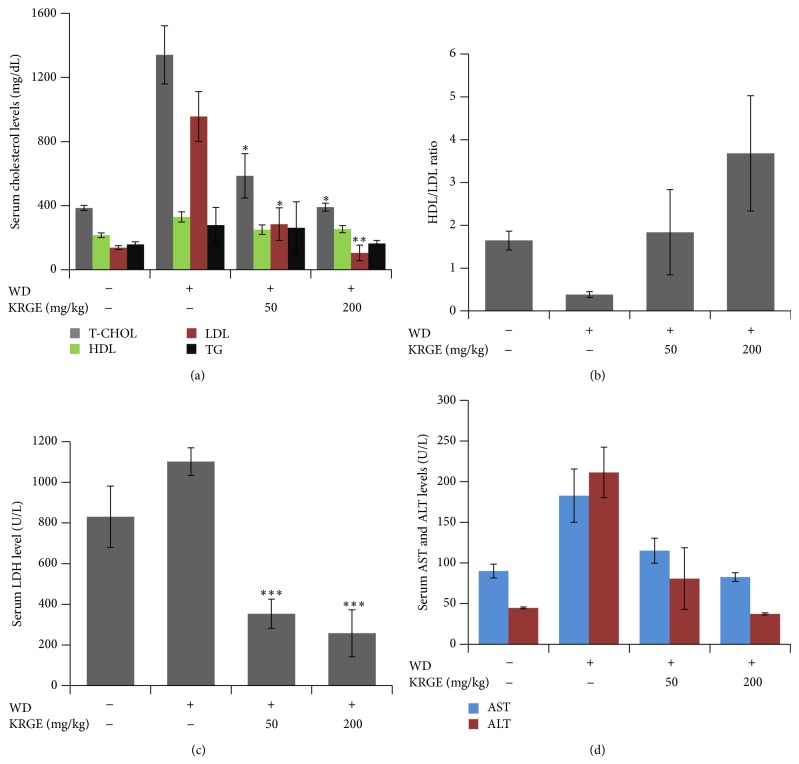
KRGE attenuated lipid profiles in LDLr^−/−^ mice. LDH, T-CHOL, HDL and TG, AST, and ALT serum levels were determined by ELISA after oral intake of KRGE (50 and 200 mg/kg/day) in mice fed with western diet. Data were presented as mean values ± SEM (*n* = 6). ^*^ *P* < 0.05, ^**^ *P* < 0.01, ^***^ *P* < 0.001 compared to control group.

To investigate the effect of KRGE on serum lipid profiles, we analyzed serum levels of total cholesterol (T-CHOL), high-density lipoprotein (HDL), low-density lipoprotein (LDL), and triglyceride (TG). The levels of T-CHOL, LDL, and TG were increased dramatically in LDLr^−/−^ mice fed with WD; however, KRGE treatments at 50 and 200 mg/kg/day showed a significant reduction in the level of lipid profiles and lactate dehydrogenase (LDH) after 13 weeks compared to the WD group (Figures 1(a)–1(c)). In contrast, serum HDL/LDL ratio significantly increased by KRG treatment in a concentration dependent manner (Figure 1(b)).

To determine the effect of KRGE on liver function, we evaluated liver function markers of aspartate aminotransferase (AST), alanine aminotransferase (ALT), and gamma-glutamyl transpeptidase (GGT) levels in the serum. KRGE diminished AST and ALT levels (Figure 1(d)), which are used as diagnostic markers for liver disease.

### 3.2. Effect of KRGE on Proinflammatory Cytokines and Adipokines in Adipose Tissue

**Figure 2:**
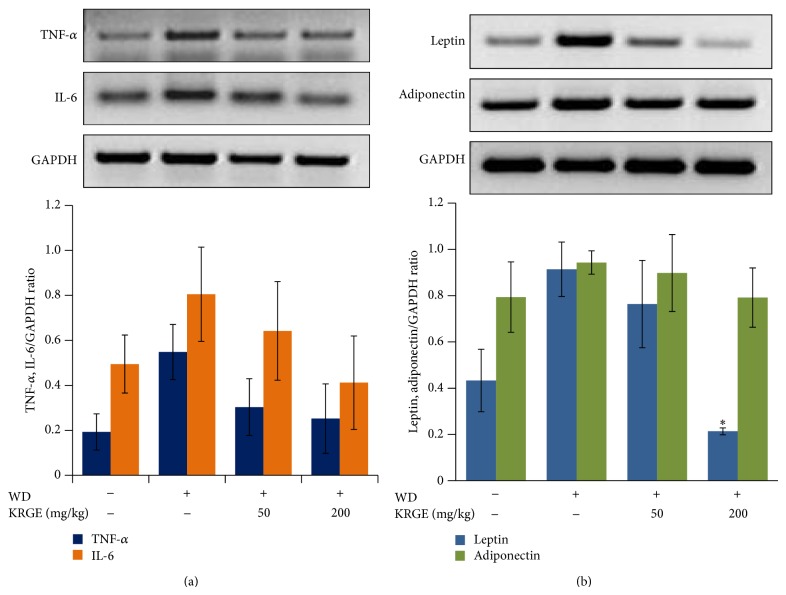
The effect of KRGE on proinflammatory mediators and adipokines in adipose tissue. The levels of TNF-*α* and IL-6 (a); leptin and adiponectin (b) were evaluated at mRNA level by RT-PCR in adipose tissue from LDLr^−/−^ mice. GAPDH was used as an internal control for RNA loading. Images represented 4 independent experiments. Values in bar graphs are mean ± SEM of at least 4 independent experiments. ^*^ *P* < 0.05 compared to control group.

Tumor necrosis factor-*α* (TNF-*α*) and interleukin-6 (IL-6) production are critical cytokines involved in atherosclerotic lesions. Both cytokines have also positive correlation with leptin expression [16, 17], an adipokine usually expressed in arthrosclerosis. Treatment with KRGE reduced the expression of TNF-*α* and IL-6 mRNA expressions in mice fed with western diet (Figure 2(a)). While increased expression level of leptin is associated with cardiovascular diseases that lead to myocardial infarction and stroke [18], decreased level of adiponectin is reported in type 2 diabetes and cardiovascular diseases [19]. To this end, we examined the effect of KRGE on leptin and adiponectin mRNA expressions. As shown in Figure 2(b), leptin mRNA expression in western diet group was significantly reduced by KRGE treatment (50–200 mg/kg/day); however, the expression of adiponectin was unaffected.

### 3.3. KRGE Inhibited Atherosclerotic Plaque Formation in Aortic Sinus of LDLr^−/−^ Mice

**Figure 3:**
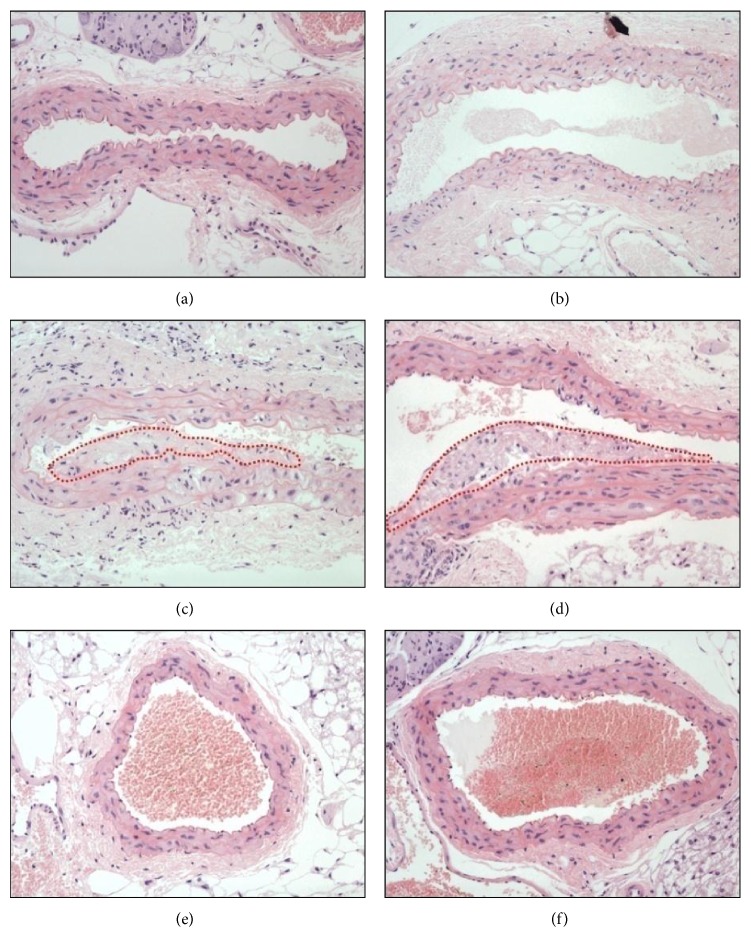
KRGE inhibited atheroma formation in aortic sinus. Mice were given normal chow diet (NCD), western diet (WD), and WD supplemented with KRGE for 13 weeks. Fixed aortic sinuses were stained with H&E. (a) NCD-fed wild type mice, (b) NCD-fed LDLr^−/−^ mice, and ((c) and (d)) WD-fed LDLr^−/−^ mice with 50 mg/kg/day (e) or 200 mg/kg/day (f) of KRGE. The dotted area exhibits lipid deposition in the aorta.

Atherosclerotic plaque formation in aortic sinus is a common phenomenon in mice fed with western diet. Therefore, we determined if treatment of KRGE (50–200 mg/kg/day) could reduce the development of plaque in aortic sinus of LDLr^−/−^ mice. As illustrated in Figure 3, endothelial thickness of the aorta in mice with a western diet (c, d) was significantly larger than those in mice with a normal diet (a, b), indicating the existence of high fat-induced atherosclerosis. However, treatment by KRGE (50 and 200 mg/kg) completely inhibited the development of atherosclerotic lesions in the cross sections of aortic valve area (e, f).

### 3.4. KRGE Inhibited Gene Expression of Proinflammatory Mediators in RAW264.7 Cells

**Figure 4:**
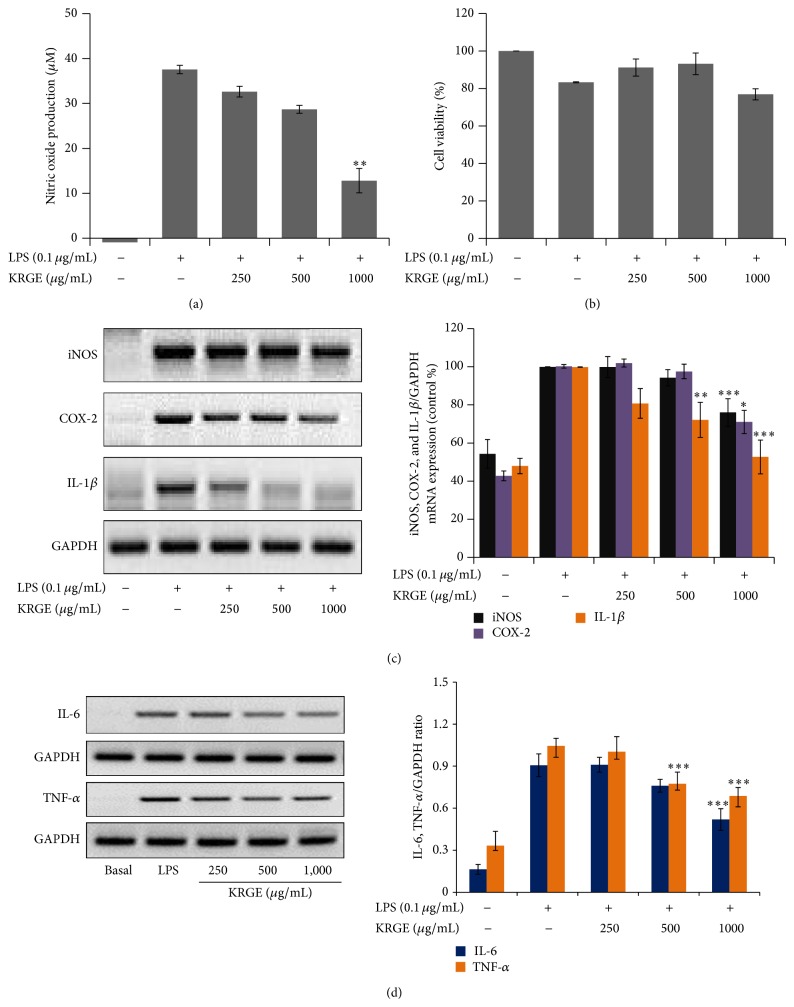
The effect of KRGE on proinflammatory mediators in LPS-activated RAW264.7 cells. RAW264.7 cells were preincubated with KRGE for 30 min and then stimulated with LPS (0.1 *μ*g/mL) for 18 h. The cell supernatant was transferred to 96 wells and mixed with Griess reagent and NO release was measured, as described in Section 2 (a). The effect of KRGE on cell viability was measured by MTT assay (b). The total RNA was isolated and mRNA expression levels were determined by RT-PCR for iNOS, COX-2, IL-1*β* (c), and TNF-*α* and IL-6 (d). GAPDH was used as a loading control. Images are represented of 4 different experiments. Values in bar graphs are mean ± SEM of 4 independent experiments. ^*^ *P* < 0.05, ^**^ *P* < 0.01, ^***^ *P* < 0.001 versus LPS control.

Proinflammatory mediators are major players in the development of arthrosclerosis. Therefore, under* in vitro* conditions, we also evaluated the effect of KRGE on the gene expression levels of proinflammatory mediators in LPS-stimulated RAW264.7 macrophages. Here, we showed that KRGE (250–1,000 *µ*g/mL) inhibited LPS stimulated nitric oxide production without any observable cytotoxicity effect (Figures 4(a) and 4(b)) and attenuated the expression of iNOS, COX-2, and IL-1*β* mRNA (Figure 4(c)). In addition, other proinflammatory cytokines, IL-6 and TNF-*α*, were suppressed by KRGE pretreatment (Figure 4(d)).

### 3.5. KRGE Upregulated the Gene Expression Level of RGS5 in RAW264.7 Cells and BV-2 Microglia Cells

**Figure 5:**
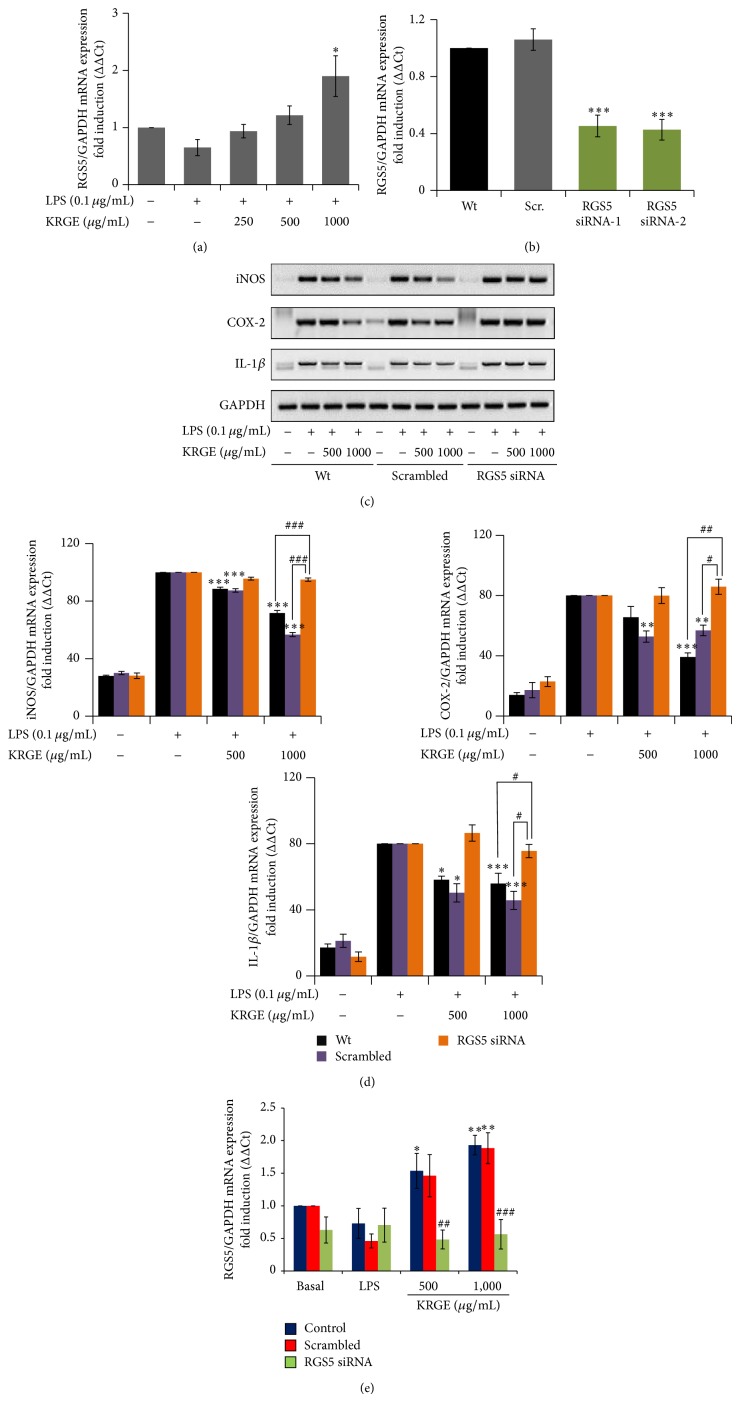
The effect of KRGE on RGS5 mRNA expression in LPS-activated RAW264.7 cells and BV-2 microglial cells. RAW264.7 cells were pretreated with KRGE for 30 min and stimulated with LPS for 18 h. RGS5 (a) mRNA expressions were determined by real time PCR. (b) BV2 microglia cells were transfected with scramble siRNA or RGS5 siRNA (100 nM) and then stimulated with LPS for 18 h to determine RGS5 mRNA expressions using RT-PCR (c) and real time PCR ((d) and (e)). The effect of RGS5 siRNA transfection was evaluated on mRNA expression of iNOS, COX-2 and IL-1*β* ((c) and (d)), and RGS5 itself (e) in KRGE treated cells by RT-PCR and real time PCR, respectively. Each bar graph represented mean ± SEM for 4 independent experiments. ^*^ *P* < 0.05, ^**^ *P* < 0.02, ^***^ *P* < 0.001, compared to LPS. ^#^ *P* < 0.05, ^##^ *P* < 0.005, ^###^ *P* < 0.001 compared to wild type or scrambled group.

RGS5 has been reported to regulate atherosclerosis, angiogenesis, and inflammation through G-protein coupled receptor (G*α*_i_ and G*α*_q_) mediated signal transductions [20]. Therefore, here we investigated whether KRGE has paramount importance in the upregulation of RGS5 mRNA expression. KRGE (250–1000 *µ*g/mL) significantly upregulated the expressions of RGS5 mRNA in LPS-activated RAW264.7 cells. We next tried to elucidate the role of RGS5 upregulation in the KRGE's anti-inflammatory activity* in vitro*. To this end, the expression of RGS5 gene was suppressed using RGS5 gene-specific siRNA by more than 70% (Figure 5(b)). Under this RGS5 knockdown condition, KRGE mediated inhibition of iNOS, COX-2, and IL-1*β* was overturned by siRNA transfection of RGS5 (Figure 5(c)). Moreover, KRGE did not induce the expression of RGS5 mRNA in RGS5 siRNA-transfected RAW264.7 cells (Figure 5(e)).

## 4. Discussion

Atherosclerosis is a pathological condition associated with increased cholesterol and triglyceride level in blood leading to stroke and coronary disease. Thus, finding a remedy and useful therapeutic target that could be used to derive an effective drug is quite important [21]. For many years, Korean red ginseng (*Panax ginseng* C. A. Meyer) has been used as traditional medicine in Asia for various ailments.* Panax ginseng* has been reported to have antiplatelet, antitumor, antioxidative, and anti-inflammatory properties [22–25]. Korean red ginseng contains many active ingredients of which ginsenosides are believed to be responsible for most of the pharmacological and immunological activities [26]. So far, many intracellular targets of Korean red ginseng and ginsenosides for various diseases have been explored; however, the antiatherosclerotic activities of Korean red ginseng through upregulation of regulator G-protein signaling remained elusive. In this study, therefore, we investigated the antiatherogenic activity of Korean red ginseng that involves RGS5 gene expression.

We found that Korean red ginseng extract (KRGE) significantly decreased the levels of total cholesterol, low-density lipoprotein, total glyceride, and lactate dehydrogenase in serum. Moreover, aspartate transaminase and alanine transaminase in the serum and leptin, adiponectin, IL-6, and TNF-*α* in adipose tissue were remarkably decreased by KRGE (Figures 1 and 2). Furthermore, the expression of proinflammatory enzymes and cytokines was attenuated in murine macrophage cells exposed to lipopolysaccharide (Figure 4). It has been reported that high lipid profile in the serum and proinflammatory mediators accelerate the development of atherosclerotic plaque formation in aortic sinus [8, 27–29]. In this regard, our work strengthens previous findings on the importance of Korean red ginseng against atherosclerosis and in particular of lipid homeostasis by downregulating proinflammatory mediators and reducing the levels of serum lipid profile. Although active ingredients of KRGE displaying antiatherosclerotic activity were not determined, we assume that anti-inflammatory activity of protopanaxadiol saponins such as ginsenoside Rb1, Rc, and Rd2 plays a major role in the prevention of atheroma formation. It has been previously reported that red ginseng saponin fraction rich in protopanaxadiol (e.g., G-Rb1, G-Rc, G-Rb2, and so on) showed anti-inflammation* in vitro* and* in vivo* [30, 31]. Moreover, KRGE directly inhibited inflammatory mediator production in LPS-activated RAW264.7 cells (Figure 4). Next, the candidate efficacy component of KRGE could be the red ginseng acidic polysaccharide (RGAP). We have reported that RGAP reduced triglyceride and nonesterified fatty acid levels in hyperlipidemic mice [32]. On the other hand, platelets signify a critical linkage between inflammation, thrombosis, and atherogenesis [33]. Thus, aberrant platelet activation and aggregation could lead to the development of atherosclerotic lesions and atherothrombosis. It is well known that ginsenoside Rg3 and its derivatives showed potent antiplatelet activity* in vitro* and antithrombosis* in vivo* [34, 35]. Taken altogether, these biological active ingredients (i.e., G-Rg3 and its derivatives) in Korean red ginseng water extract possibly play a pivotal role in the prevention of atherosclerosis.

Regulator of G-protein signaling (RGS) proteins plays critical role in the regulation of G-protein coupled receptor signaling pathway by binding to the active G subunits and stimulating GTP hydrolysis. RGS5 can switch off G-protein mediated signaling pathway [36], thereby preventing cardiac hypertrophy, atherosclerosis, and angiogenesis [37, 38]. Considering the antiatherosclerotic importance of ginseng, we investigated if the extract of this plant modulates mRNA expression of RGS5 in BV-2 microglial cells. We found that KRGE upregulated mRNA expression of RGS5 and RGS5 siRNA transfection reversed the inhibitory effect of KRGE on iNOS, COX-2, and IL-1*β* mRNA expression (Figure 5), suggesting that the anti-inflammatory effect of Korean red ginseng could be partially mediated by RGS5 signaling.

## 5. Conclusion

In conclusion, Korean red ginseng extract inhibited serum total cholesterol and triglyceride in western diet-induced atherosclerosis in LDL receptor gene deleted-mice. In addition, Korean red ginseng extract ameliorated the atherosclerotic plaque in mice. On the other hand, Korean red ginseng extract impaired LPS-induced inflammatory mediator production in RAW264.7 cells. Besides, Korean red ginseng extract increased the expression of RGS5 mRNA in a concentration-dependent manner. Our finding revealed the antiatherosclerosis of Korean red ginseng extract may involve RGS5 signaling which could also be the possible intracellular target against chronic inflammation prone to atherosclerosis.

## Figures and Tables

**Table 1:** The sequence of oligonucleotides.

Gene	Primer	Oligonucleotide sequence (5′-3′)
GAPDH	F	5′CAATGAATACGGCTACAGCAAC3′
	R	5′AGGGAGATGCTCAGTGTTGG3′

iNOS	F	5′CCCTTCCGAAGTTTCTGGCAGCAGC3′
	R	5′GGCTGTCAGAGCCTCGTGGCTTTGG3′

COX-2	F	5′CACTACATCCTGACCCACTT3′
	R	5′ATGCTCCTGCTTGAGTATGT3′

IL-1*β*	F	5′CAGGGTGGGTGTGCCGTCTTTC3′
	R	5′TGCTTCCAAACCTTTGACCTGGGC3′

TNF-*α*	F	5′TTGACCTCAGCGCTGAGTTG3′
	R	5′CCTGTAGCCCACGTCGTAGC3′

IL-6	F	5′GCTGGAGTCACAGAAGGAGTGGC3′
	R	5′GGCATAACGCACTAGGTTTGCCG3′

Leptin	F	5′TGAACAAAGGGGCTTGGGTT3′
	R	5′TGTGCCCTGAAATGCGGTAT3′

Adiponectin	F	5′GCTACTGTTGCAAGCTCTCCT3′
	R	5′TCGTAGGTGAAGAGAACGGC3′

RGS5	F	5′CCCAAGGAGTGAACCGGCTGT3′
	R	5′GCACTGCCCTTGAGGCACCC3′

## Abbreviations

KRGE: Korean red ginseng water extract
RGS 5: Regulator of G-protein signaling 5
LDH: Lactate dehydrogenase
TC: Total cholesterol
TG: Triglyceride
NO: Nitric oxide
IL-6: Interleukin6
TNF-*α*: Tumor necrotic factor alpha
COX-2: Cyclooxygenase-2
CVD: Cardiovascular diseases
ICAM-1: Intracellular adhesion molecule-1
MCP-1: Macrophage chemoattractant protein-1.
